# Industrial Two-Phase Olive Pomace Slurry-Derived Hydrochar Fuel for Energy Applications

**DOI:** 10.3390/polym16111529

**Published:** 2024-05-29

**Authors:** Adnan Asad Karim, Mᵃ Lourdes Martínez-Cartas, Manuel Cuevas-Aranda

**Affiliations:** 1 Department of Chemical, Environmental and Materials Engineering, Science & Technology Campus (Linares), University of Jaén, Avda. de la Universidad s/n, 23700 Linares, Spain; mcuevas@ujaen.es; 2 University Institute of Research on Olive and Olive Oils (INUO), University of Jaén, Campus de las Lagunillas s/n, 23071 Jaén, Spain

**Keywords:** olive pomace, hydrothermal process, hydrochar fuel, combustion, bioenergy

## Abstract

The present study aims to resolve the existing research gaps on olive pomace (OP) hydrochars application as a fuel by evaluating its molecular structures (FTIR and solid NMR analysis), identifying influential characteristics (Pearson correlation analysis), process optimization (response surface methodology), slagging–fouling risks (empirical indices), and combustion performance (TG-DSC analysis). The response surfaces plot for hydrothermal carbonization (HTC) of OP slurry performed in a pressure reactor under varied temperatures (180–250 °C) and residence times (2–30 min) revealed 250 °C for 30 min to be optimal conditions for producing hydrochar fuel with a higher heating value (32.20 MJ·Kg^−1^) and energy densification ratio (1.40). However, in terms of process efficiency and cost-effectiveness, the optimal HTC conditions for producing the hydrochar with the highest energy yield of 87.9% were 202.7 °C and 2.0 min. The molecular structure of hydrochar was mainly comprised of aromatic rings with methyl groups, alpha-C atoms of esters, and ether bond linkages of lignin fractions. The slagging and fouling risks of hydrochars were comparatively lower than those of raw OP, as indicated by low slagging and fouling indices. The Pearson correlation analysis emphasized that the enrichment of acid-insoluble lignin and extractive contents, carbon densification, and reduced ash content were the main pivotal factors for hydrochar to exhibit better biofuel characteristics for energy applications.

## 1. Introduction

Bioenergy is a renewable energy resource, and its intensive global usage is necessary to achieve net zero emissions by 2050 (1.5 °C scenario) and affordable clean energy under UN sustainable development goals. It is forecast to contribute about 22% of the global total supply of primary energy by 2050 [1]. Boosting the utilization of biofuels derived from waste lignocellulosic biomass is essential to accomplishing bioenergy targets [2]. In this context, hydrochar has emerged as a promising solid biofuel due to its high energy density, calorific value, and combustion performance [3]. It is mainly produced by hydrothermal carbonization of lignocellulosic biomass waste with high water content, such as sewage sludge, food waste, algae, animal manure, etc. [4,5]. Compared to other treatment methods, i.e., dry pyrolysis and gasification, this method provides several advantages due to the elimination of the drying step, its low temperature (180–250 °C), and its ability to process wet biomass directly. This makes the HTC process for hydrochar more beneficial and energy efficient [6,7,8]. However, uneven geographical and local availability of biomass is one of the major challenges for realizing industrial production of hydrochar. Therefore, to make hydrochar production more sustainable and practically feasible, it is crucial to study the production of hydrochar from specific biomass on the basis of its geographical distribution for biofuel application. 

Spain is the top-most global producer of olive oil, with an annual production of 1.36 million metric tons reported for the 2021–2022 year [9]. It is produced by the olive oil industry mainly through a two-phase system, which involves the crushing and separation of oil from olives [10]. In this process, each ton of olives generally produces approximately 20% oil and 80% olive pomace (OP) slurry [11]. This means OP is one of the most abundantly available waste biomasses in Spain [12] and, hence, could be an ideal feedstock for hydrochar production. The physicochemical characteristics of OP vary due to the diverse chemical composition of olive fruit, which is influenced by agroclimatic factors and cultivation conditions [13,14,15]. The industrial two-phase OP mainly consists of olive pulp and seeds. It also has a high amount of water (60–80%), contains phytotoxic compounds (polyphenols), and is possibly contaminated with pesticides [16,17,18], which makes its disposal and storage very challenging [19,20]. To resolve this challenge, the HTC of OP to produce hydrochars for biofuel applications was recommended by several research studies. These studies were conducted using OP with varied chemical composition and under different HTC conditions, i.e., 180–300 °C temperature, 0–24 h residence times, and 1/10–1/2 biomass–water ratio. Consequently, hydrochars exhibited a heterogeneous composition but similar fuel characteristics [13,18,19,20,21,22]. 

The advancement of research on OP hydrochar is essential to promoting its practical applications as a biofuel [23,24,25]. For instance, the mineral composition of hydrochars and associated slagging and fouling risks have seldom been investigated [26]. The underlying mechanism for OP hydrochar formation by the HTC is not known properly. It is also not identified which properties of OP hydrochar are decisive for increasing its calorific value and energy density. The combustion behavior of OP hydrochar has also been scarcely reported, which is crucial for its potential energy application. Furthermore, in the literature, most of the research studies have focused on the exhausted OP (a solid residue obtained after recovery of OP oil). Production of exhausted OP involves energy-intensive and expensive transportation, storage, drying, and solvent extraction steps [20]. It has been mainly used locally as a biofuel for the production of heat and electricity. However, its combustion could release fine particles and toxic chemicals (e.g., dioxins and furans) into the environment, which could be harmful to human health [18,27,28,29]. Research on the direct conversion of industrial two-phase OP slurry into hydrochars for energy applications is rarely investigated. The present work suggests an alternative non-conventional approach for promoting in situ and decentralized use of two-phase OP in the olive oil industry to produce hydrochar for biofuel applications. 

The originality and specific objectives of the present work are (1) determining the molecular structural characteristics of two-phase OP hydrochars by FTIR and solid NMR analysis; (2) performing Pearson correlation analysis to identify the specific properties essential to producing highly energy-dense hydrochar; (3) evaluating the slagging and fouling risks for the OP and its hydrochars; (4) assessing the optimum HTC conditions on the basis of response surface methodology to produce hydrochar with the most appropriate fuel characteristics; and (5) studying the combustion performance of the raw OP and optimal hydrochar. 

## 2. Materials and Methods

### 2.1. Raw Material

The two-phase OP (*Picual* sp.) slurry (80.43 ± 0.17% of water content) was procured from Almazara Andrés Aguilar (Olive Oil Industry), Linares, Jaen, Spain, with UTM coordinates: 38°04′26.86″ N, 3°38′52.31″ W. On an oven-dried basis, the solid two-phase OP consisted of 10.82% cellulose and 28.30% hemicellulose, as estimated by the Van Soest et al. (1991) method [30].

### 2.2. Experiments for Hydrochar Production

The HTC of OP was performed in a pressure reactor (Parr 4848, Parr Instrument Company, Moline, IL, USA) to produce hydrochars based on the central composite design (CCD) of the response surface methodology. Table S1 (Supplementary Information) presents the coded and real values of independent variables for experimental CCD. Experiments were undertaken with two process variables, i.e., temperature (180–250 °C) and holding time (2–30 min). These process conditions were adapted on the basis of the previous research findings [23,31]. The stirrer speed was 150 rpm, while the heating rate was approximately 2 to 5 °C·min^−1^, and the cooling rate was from 10 to 20 °C·min^−1^ for all the HTC experiments. The maximum autogenous pressure recorded during the experiments at different HTC temperatures was around 14 bars (180 °C), 32 bars (215 °C), and 70 bars (250 °C). The hydrochars were separated from the slurry by using filter paper (Cod. RM13054252, Filter Lab, Barcelona, Spain) and the vacuum filtration technique, followed by air-drying to achieve a uniform moisture content for long-term storage in sealed plastic bags. Abbreviations used for hydrochar samples were 180-2, 180-16, 180-30, 215-2, 215-16, 215-30, 250-2, 250-16, and 250-30. For instance, 180-2 represents hydrochar created at 180 °C with a 2 min holding time.

Modde 6.0 software (Umetric AB, Umeå, Sweden) was used to prepare CCD for HTC trials and subsequent data analysis on the basis of a second-order polynomial model (Equation (1)) involving two independent variables (temperature and residence time) and three responses (hydrochar yield, energy densification ratio, and energy yield).

*Y* = *β*_0_ + (*β*_1_·*T*_R_) + (*β*_2_·*t*_R_) + (*β*_3_·*T*_R_·*T*_R_) + (*β*_4_·*t*_R_·*t*_R_) + (*β*_5_·*T*_R_·*t*_R_) (1)

where *Y* is the response value, *T*_R_ and *t*_R_ are the coded values of the two independent variables, and *β_i_* (*i* = 1–5) are the intercept term (*β*_0_), the linear effects (*β*_1_ and *β*_2_), the squared effects (*β*_3_ and *β*_4_), and the interaction effects (*β*_5_) calculated from the experimental data by regression analysis. The experimental data validation was performed by a one-way ANOVA statistical test with a 95% confidence level.

### 2.3. Characterization of Raw Material and Hydrochar

Hydrochar yields (%) were quantified on the basis of oven-dried OP biomass and hydrochars [32]. Acid-insoluble lignin (AIL) content was estimated by thermally treating the samples in a 72% H_2_SO_4_ solution (TAPPI-t-222-OS-74 method) [33]. Extractive (E) content was estimated using the hexane solvent-based extraction method [34]. Moisture (M) content was measured by oven-drying samples at 105 °C until a constant weight was obtained. Volatile matter (VM) was measured gravimetrically by heating samples in a closed crucible at 900 ± 25 °C [35,36]. Ash content was determined by heating the samples in an open crucible at 575 ± 25 °C for 5 h (NREL/TP-510-42622 method) [37]. The fixed carbon (on a dry basis) was indirectly calculated by subtracting the VM and ash contents from 100 (Equation (2)). The CHNS elemental analyzer (TruSpec Micro, Leco Corporation, St. Joseph, MI, USA) was used to quantify total carbon (C), hydrogen (H), nitrogen (N), and sulfur (S) content. Oxygen (O) content was measured using Equation (3), as per ASTM E870-82 method [38]. The fuel ratio indicates the combustibility potential of hydrochar, which was estimated by dividing the FC and VM contents of the hydrochars [23]. The fixed carbon index [39,40] and carbon densification factors [13] were calculated by Equations (4) and (5). The higher heating value was measured using a bomb calorimeter (Parr Series 6400, Parr Instrument Company, Moline, IL, USA). The energy densification ratio (EDR) of samples was determined using Equation (6) [23]. Energy yield (EY), also known as energy recovery efficiency, was calculated by multiplying hydrochar yield by EDR [41]. Analysis of surface functional groups present in samples was conducted by Fourier transform infrared spectroscopy (Vertex 70 spectrophotometer, Bruker, Billerica, MA, USA) in the wavelength range of 400–4000 cm^−1^ with 4 cm^−1^ resolution and 100 scans. The NMR spectrophotometer (Bruker AVANCE, Billerica, MA, USA) was utilized for solid-state ^13^C NMR analysis of samples with a 4 mm CP-MAS probe performed at 100.62 MHz, 2 ms contact time, and 1200 scans. The NMR spectra were analyzed using the TopSpin 3.6.5 software package (Academic license).

(2) $$FC\left(\%\right)=100-\left(M\%+VM\%+Ash\%\right)$$

(3) $$O\left(\%\right)=100-\left(C\%+H\%+N\%+S\%+Ash\%\right)$$

(4) $$FCI=\frac{{FC}_{Hydrochar\;at\;t{}^{\circ}C}-{FC}_{raw\;material}}{{FC}_{Hydrochar\;at\;250\;{}^{\circ}C}}$$

(5) $$CDF=\frac{{\%C\;}_{dried\;hydrochar}}{{\%C\;}_{dried\;olive\;pomace}}$$

(6) $$EDR=\frac{{HHV}_{dried\;hydrochar}}{{HHV}_{dried\;olive\;pomace}}$$

### 2.4. Slagging and Fouling Risks Evaluation

The oven-dried OP biomass and hydrochar were digested using a mixture of HNO_3_ and H_2_O_2_ at 140 °C, 15 min holding time, and 1000 W energy in a microwave digestion system (ETHOS One, Milestone, Sorisole, Italy) to extract metals and their quantification by ICP-OES analysis. The metal oxides were measured by multiplying the total metal contents with conversion factors [42]. A variety of empirical indicators, including the alkali index, base-to-acid ratio, slagging index, fouling index, slag viscosity index, and bed agglomeration index, were used to assess the slagging and fouling risks of raw OP biomass and hydrochars [43,44]. Table 1 shows the procedures used to compute the slagging and fouling indices. The alkali index (AI) of a fuel is an indicator of slagging–fouling propensities, which was estimated using alkali oxides per heat unit (Kg·GJ^−1^). The base-to-acid ratio (B/A) reflects the ash fusion temperature and slag viscosity. It was calculated based on the concentrations of the basic (Fe_2_O_3_, CaO, MgO, Na_2_O, K_2_O, and P_2_O_5_) and acidic (SiO_2_, Al_2_O_3_, and TiO_2_) minerals. The slagging index (SI) forecasts the propensity for fused slag deposition on furnace walls by considering the sulfur concentration and base-to-acid ratio. The tendency for corrosive alkali minerals to build on the furnace walls is predicted by the fouling index (FI). The slag viscosity index (SVI) predicts the slagging tendency of the metal oxides by considering the silica concentration and the probable creation of metal silicates with low melting temperatures. Metal oxide agglomeration in combustion furnaces is forecasted by the bed agglomeration index (BAI). A higher risk of bed agglomeration is indicated by a fuel BAI value of less than 0.15 [43]. 

### 2.5. Combustion Performance Analysis of Raw Material and Hydrochar

An oxidative environment was used to assess the samples’ combustion behavior using a thermogravimetric (TG) analyzer (TDA/SDTA 851e, Mettler Toledo, Columbus, OH, USA) and a differential scanning calorimeter (DSC 822e, Mettler Toledo, Columbus, OH, USA). The analysis was carried out with an air flow rate of 150 mL·min^−1^, a temperature range of 25–900 °C, and a heating rate of 10 °C·min^−1^. The T_i_ (ignition temperature) and T_b_ (burnout temperature) were determined from the DTG profiles. T_i_ is defined as the temperature where the weight loss rate is 1%·min^−1^ after the first weight loss due to the presence of residual moisture; T_b_ is defined as the temperature value at which the DTG reaches 1%·min^−1^ at the end of the curve [45,46]. 

## 3. Results

### 3.1. Molecular Structure and Formation of Hydrochar

**Figure 1:**
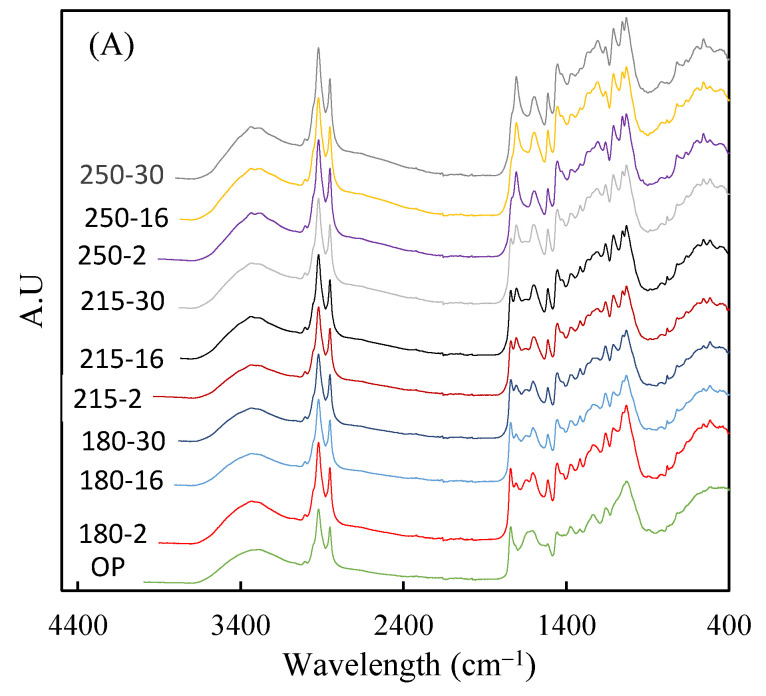

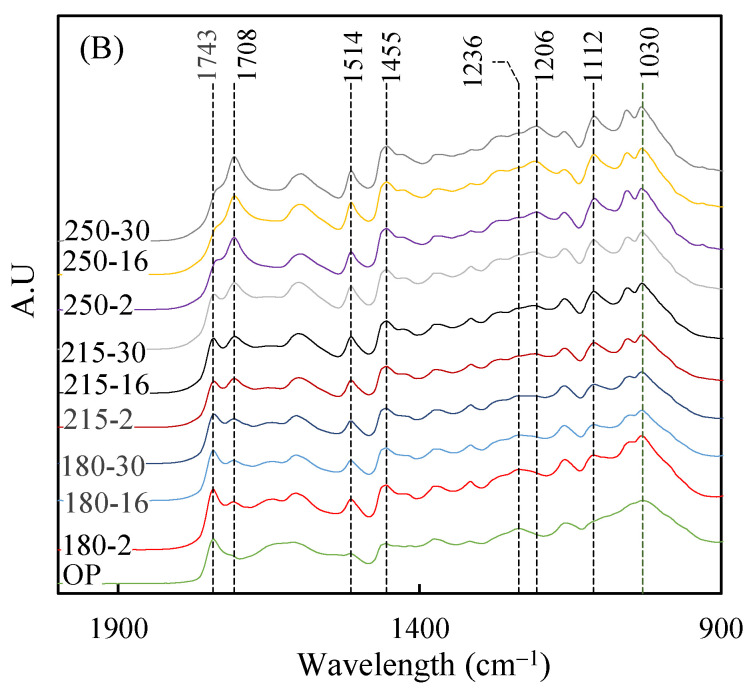
Infrared spectra for raw material and wet olive pomace hydrochars, (**A**) 400–4000 cm^−1^ region, and (**B**) 900–2000 cm^−1^ region.

FTIR analysis enables the visualization of changes that hydrothermal treatments induce in the surface functional groups of biomasses. In Figure 1, FTIR spectra are presented for both the raw material and the hydrochars generated at different temperatures and reaction times. Figure 1A displays spectra in the wavelength range of 400–3500 cm^−1^, revealing a broad band centered at 3300 cm^−1^, which has frequently been associated with vibrations in O-H bonds [47]. This signal could arise from both the water present in samples and bonds within functional groups such as hydroxyl and carboxyl. Additionally, all spectra exhibit two peaks, located around 2920 cm^−1^ and 2850 cm^−1^, which have been associated with C-H bonds in aliphatic and aromatic structures [48]. These signals tend to intensify with increasing temperature and reaction time, which could be explained by the increase in lignin percentage in the hydrothermally treated biomasses.

The wavelength region where the hydrothermal treatment caused the most variations in spectral signals is between 900 cm^−1^ and 1900 cm^−1^. Therefore, this region is detailed in Figure 1B, where it is observed that an increase in treatment severity led to a decrease in the intensity of the spectral peaks at 1236 cm^−1^ and 1743 cm^−1^. These two signals have been related to the stretching vibrations of C=O bonds in carbonyl or acetyl groups and the C-O-C bonds of acetyl groups present in hemicelluloses [49,50]. This indicated that the hydrothermal treatment produces strong hydrolysis of hemicellulosic polymers. However, the treatment likely did not achieve complete elimination of the cellulose fraction since the signals at 1206 cm^−1^ and 1030 cm^−1^, related to this polymer [51,52], did not weaken considerably (Figure 1B). On the other hand, an increase in treatment severity led to a clear increase in the spectral signal centered at 1708 cm^−1^, which could be the result of condensation reactions (repolymerization) between degradation products from carbohydrates and lignin. This signal has been highlighted previously in hydrothermal treatments applied to almond-tree pruning [53] and olive-fruit endocarps [46]. Figure 1B also shows increases in spectral signals related to lignin, such as those centered at 1514 cm^−1^, 1455 cm^−1^, and 1112 cm^−1^, which have been associated with aromatic rings, C-H deformation in methyl and methylene groups, and aromatic C-H deformation of syringyl units [52,54].

**Figure 2:**
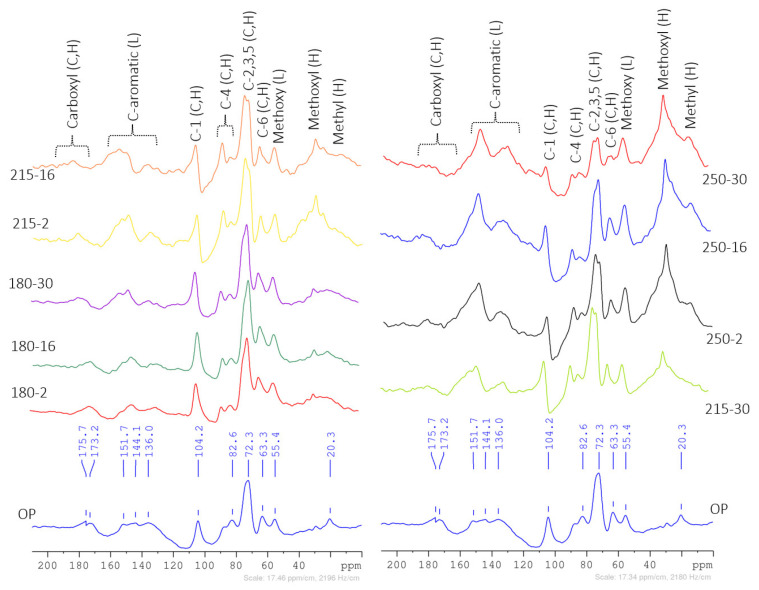
^13^C solid NMR analysis of raw materials and hydrochars.

The ^13^C solid NMR spectra (Figure 2) showed the varied molecular structures of raw materials and hydrochars. Peak’s areas attributed to cellulose, hemicellulose, and lignin were estimated through deconvolution (mixed Lorentzian and Gaussian techniques) [55], presented in Tables S2–S11 (Supplementary Information). This was used to envision the relative quantitative change in the molecular groups [56,57] probably caused by hydrothermal carbonization. Peak interpretations were performed based on the literature on lignocellulosic biomass composition analysis by ^13^C NMR spectra [58,59]. The peak signals of carboxyl groups (165–190 ppm) were not identified in the hydrochars produced from 215-30 onwards due to their loss through decarboxylation reactions. Aliphatic methyl groups (10–25 ppm signals) are present at different signals in the OP (20 ppm) and hydrochars (13 ppm). Peak signals (30–50 ppm) attributable to alkyl groups of hemicellulose fractions were not found in the 250-30 hydrochar. This indicated a complete loss of hemicellulose fractions due to its depolymerization achieved at 250-30 HTC conditions. Acetal C atoms of holocellulose, identifiable by peak signals in 90–110 ppm, were found to be least affected by the HTC between 180-2 and 250-16, but their area was only enhanced in 250-30. The area of peaks attributable to crystalline (89–91 ppm) and amorphous (84–86 ppm) cellulose was prominently reduced by HTC from 250-16 onwards. Prominent changes in the range (110–140 ppm) of peak signals, such as 135 ppm peaks not observed in hydrochars (250-2 to 250-30) and a new 130 ppm peak (not identified in the OP) indicative of arenes and phenols of lignin observed in 180-16 onwards hydrochars, and its area increased with HTC process severity elevation. These changes were indicators of depolymerization and subsequent aromatization reactions (new molecular arrangement), causing the formation of modified aromatic and heteroaromatic moieties compared to raw OP.

Peak signals (72 ppm, 71 ppm, and 63 ppm) associated with an overlapping region of holocellulose (hydroxyl-substituted C atoms of holocellulose) and lignin (Sp^3^-hybridized C atoms bonded with hydroxyl or oxygen in esters) were identified. The peak area of 72 ppm gradually decreased with an increase in the HTC process severity. The 71 ppm peak signal was new and only found in the spectra of hydrochars; its area was substantially decreased in the 250-30 HTC condition. This could reflect that this peak was associated with new (not present in OP) aromatic moieties formed through polycondensation and aromatization reactions [60] and their reduction with intensified HTC severity. The peak areas of 20–30 ppm signals of methyl groups bonded to aromatic rings and 55 ppm of methoxy groups increased with HTC severity in hydrochars. A substantial increase in the area of peak signals (140–165 ppm) was observed, with an increase in HTC severity (180-2 to 250-30) attributed to the syringyl and guaiacyl groups of lignin. Furthermore, an area of 63 ppm associated with aromatic groups exhibited narrow differences, which could indicate the thermo-resistant nature and retention of insoluble lignin fractions in hydrochars. 

The above results indicated the following possible mechanisms for the formation of hydrochar: The HTC under subcritical conditions (hot compressed water and autogenic pressure) involved primary and secondary-stage reactions to form hydrochars. The primary stages included hydrolysis, dehydration, depolymerization, deoxygenation, decarboxylation, and demethylation, which generally occurred simultaneously [61]. These reactions weakened the polymeric structures of hemicellulose, cellulose, and lignin. In the initial stage of HTC (at 180 °C), the hemicellulose hydrolyzes and depolymerizes into soluble monosaccharides, oligosaccharides, and other intermediates (organic acids, furfurals) in the liquid phase [62]. The hydrolysis (above 150 °C) facilitated the depolymerization of hemicellulose, which occurs at a reaction temperature of 180–200 °C in the OP [63]. The dehydration and decarboxylation reactions also occurred, which were established by the lower H/C and O/C ratios of hydrochars [61,64]. The cleaving of β-(1-4)-glycosidic bonds in cellulose resulted in the formation of soluble oligosaccharides, glucose, and furfurals. In this case, amorphous fractions of cellulose were mostly affected above 210 °C, while the crystalline part was least affected and mainly cleaved above 230 °C. The soluble fractions of lignin decomposed into phenolic derivatives. 

The secondary stage consisted of repolymerization and aromatization reactions happening progressively with an increase in temperature and residence times [61]. Repolymerization reactions involved aldol condensation (α-carbonyl aldehydes), acetal cyclization, and etherification to form aromatic phenolic structures [65]. The insoluble part of lignin becomes enriched on a mass basis in hydrochar due to its thermo-resistant molecular structure (mainly polyaromatic hydrocarbons) and the loss of other aliphatic structures. Due to the HTC, major changes were found to be the loss of hydroxyl, carboxyl, and methyl groups from the OP biomass. The molecular structure of 250-30 hydrochar was found to consist of predominantly aromatic rings with methyl groups, alpha-C atoms of esters, Sp^3^—hybridized C atoms bonded with oxygen in ethers of lignin fractions, and aliphatic methyl and acetal groups of carbohydrates. Earlier studies also reported that the hydrochar mainly consisted of aromatic molecular structures (polyaromatic hydrocarbons) and aliphatic fragments [62,65,66,67].

### 3.2. Characteristics of Raw Material and Hydrochars

The mass yield, AIL, E, and proximate characteristics of hydrochars are presented on an oven-dried basis in Table 2. In addition, the ultimate and energetic characteristics of hydrochars are shown in Table 3. An increase in HTC process severity resulted in changes in hydrochar characteristics. The yield (50.19–77.34%), ash (2.78–4.23%), VM (69.35–77.91%), and O contents (27.26–15.26%) of hydrochars decreased with the increase in the severity of the treatment. This trend was due to the increased dehydration, depolymerization, deoxygenation, and transfer of soluble fractions (e.g., minerals) present in OP into liquid fractions [62].

The values of AIL (58.54–76.01%), E (16.00–24.67%), FC (17.92–27.87%), fuel ratio (0.23–0.40%), C (59.80–71.83%), and HHV (24.62–32.20 MJ·Kg^−1^) in the hydrochars were enhanced. The AIL and HHV increased by 2.06 and 1.24 times, respectively, while the ash and VM contents were reduced by 0.65 and 0.86 times, respectively. The FCI and CDF, which are indicators of carbon enrichment in hydrochars, were increased from 0.09 to 0.45 and from 1.04 to 1.25, respectively. Furthermore, the EDR of hydrochars increased from 1.07 to 1.40. Overall, the hydrochar produced at 250 °C for 30 min exhibited relatively better fuel characteristics in terms of HHV (32.20 MJ·Kg^−1^), fuel ratio (0.40), and EDR (1.40). Under similar conditions, the HHV and EDR of this OP hydrochar were observed to have very narrow differences from earlier reported values in the literature. Gimenez et al. (2020) reported a maximum HHV value of 32.64 MJ·Kg^−1^ and 1.61 EDR for hydrochar produced from two-phase olive pomace slurry at 240 °C and 6 h residence time [13]. Micali et al. (2019) explored the hydrothermal carbonization of olive pomace at process conditions of 260–305 °C and 60–180 min, and the biomass/water ratio ranged from 1/6 to 1/4. The optimal condition was reported to be 280 °C for 180 min—1/6 producing hydrochar with a 31.14 MJ·kg^−1^ calorific value [21]. 

The EY is a commonly used parameter for identifying the suitable HTC conditions with the lowest energy consumption that produce hydrochar with maximum energy retention. This parameter helps to increase the energy efficiency and economic feasibility of the production process [68,69]. In the present study, the hydrochars produced at 215 °C for 2 min showed a relatively higher EY of 88.26%, compared to 70.49% at 250 °C for 30 min. 

**Figure 3:**
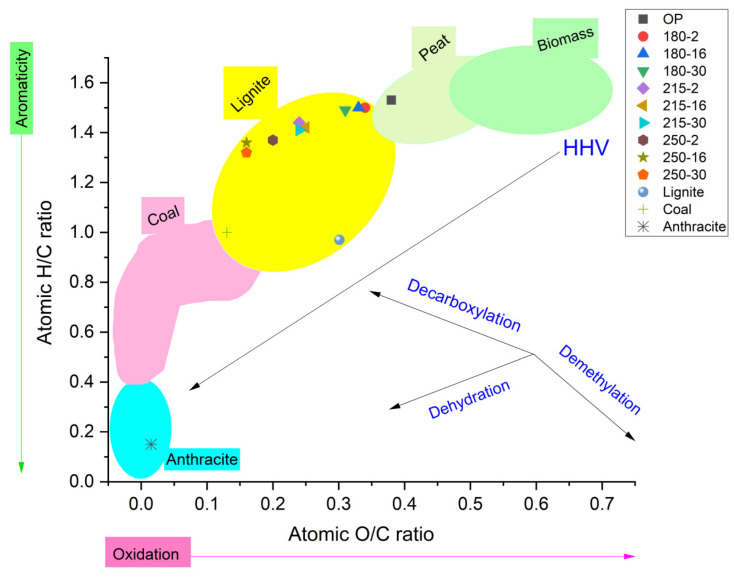
van Krevelen plot for raw materials and hydrochars.

The van Krevelen diagram (Figure 3) showed that the increase in HTC process severity reduction in H/C and O/C ratios was caused by the intensified dehydration and decarboxylation reactions during the HTC of OP [13,70]. Consequently, hydrochar produced at 250 °C for 30 min possessed enhanced aromaticity and resembled the characteristics of lignite coal [71], which is in line with previous studies on OP hydrochar [23,31]. 

**Figure 4:**
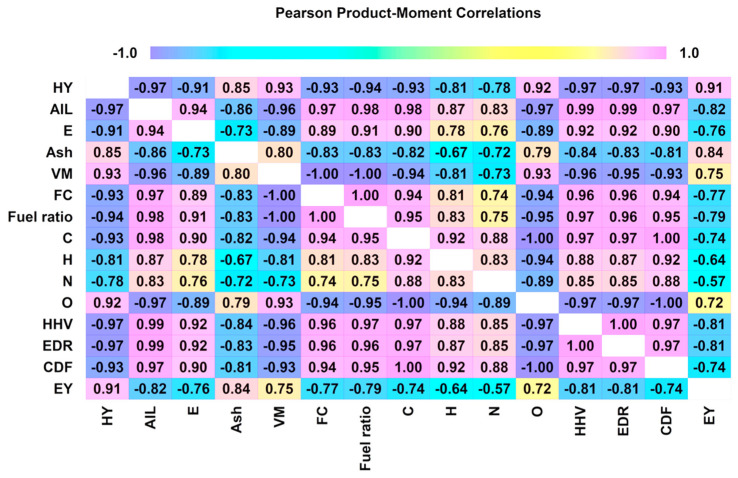
Pearson correlation plot for hydrochar characteristics.

The correlation between hydrochar characteristics was depicted in the Pearson correlation plot (Figure 4), prepared by Statgraphics Version 19.6.02 (Statgraphics Technologies, Inc., The Plains, VA, USA). The yield of hydrochar was found to be positively correlated with VM (R^2^ = 0.93) and O (R^2^ = 0.92) contents. Furthermore, HHV and EDR were observed to be positively correlated with AIL (R^2^ = 0.99), E (R^2^ = 0.92), FC (R^2^ = 0.96) and C (R^2^ = 0.97) contents. Similarly, the fuel ratio was positively correlated with AIL (R^2^ = 0.98), E (R^2^ = 0.89), C (R^2^ = 0.95), HHV (R^2^ = 0.97), EDR (R^2^ = 0.96) and CDF (R^2^ = 0.98) values. These results exhibited that to produce hydrochar with enhanced fuel characteristics (HHV, EDR), it is essential to improve the AIL, E and CDF values and reduce the VM and O contents [44]. Higher levels of lignin and extractives raise the heating value of hydrochar, which makes it a desirable property for usage in biofuel applications [72,73].

### 3.3. Slagging and Fouling Risks of Raw Materials and Hydrochars

The slagging and fouling deposition that occurs during the combustion of a fuel (like coal) deteriorates the boiler’s efficiency and life span. Thus, risk estimation for slagging and fouling is necessary for the effective application of fuel in boilers. These risks are directly related to the inorganic chemical composition (alkaline minerals) of the fuel [74]. Therefore, the mineral composition of raw materials and hydrochars was determined to measure their slagging and fouling indices (Table 4). The raw OP consisted of 2.41% K_2_O, 0.64% CaO, 0.28% Na_2_O, 0.18% P_2_O_5_, 0.15% MgO, 0.12% SiO_2_, 0.11% Fe_2_O_3_, and 0.06% Al_2_O_3_. Comparatively, the HTC high severity (250-30) caused a relative increase in SiO_2_ (0.17%), P_2_O_5_ (1.06%), and Al_2_O_3_ (0.08%) contents but contrastingly reduced the Na_2_O (0.07%), MgO (0.09), Fe_2_O_3_ (0.06%), and K_2_O (0.49%) in the hydrochar. In addition, the CaO (0.81%) content was relatively higher in hydrochars than raw OP.

In terms of slagging–fouling indices, the raw OP showed 1.17 kg·GJ^−1^ AI, 20.59 B/A, 0.21 SI, 55.43 FI, and 12.07 SVI. Comparatively, the high-severity hydrochar (250-30) exhibited reduced AI (0.17 kg·GJ^−1^), B/A (10.53), SI (0.11), and FI (5.91), but higher SVI values. Thus, the HTC of OP decreased AI, B/A, SI, and FI by 86.53%, 76.89%, 48.87%, and 96.07%, respectively. Earlier studies also reported similar results. Zhu et al. (2018) found hydrothermal carbonization (240 °C for 60 min) of corn stalk (AI = 0.9 kg·GJ^−1^; produced hydrochar with a lower AI of 0.17 kg·GJ^−1^ [75]. The AI values of hydrochar are below 0.34 kg·GJ^−1^, which indicates a lower risk of slagging–fouling. Lin et al. (2015) demonstrated hydrothermal carbonization (270 °C for 30 min) of paper sludge produced hydrochar with reduced SI (0.70) and FI (0.52), compared to raw paper sludge (SI = 1.30 and FI = 1.15) [76].

An increase in HTC temperature and residence time resulted in a decrease in the AI, B/A, SI, and FI but elevated the SVI and BAI due to the relative enrichment of SiO_2_ in the hydrochars. The AI was minimized due to a decrease in the alkali metal concentrations combined with improved HHV of hydrochars. Similarly, the SI value reduction was because of negligible S content and a lower B/A ratio—acidic oxide retention and alleviated basic oxide concentrations. In contrast to this trend, SVI increased by 75.11% in hydrochar due to the enrichment of Si compared to Ca, Mg, and Fe concentrations in the hydrochars. The SVI improvement specified a reduction in the slagging risk. This poses a risk for the application of hydrochars in boilers because Si, Na, and K can form eutectics with low initial deformation and melting temperatures. Ca, Mg, and Fe can react with Si to form silicate minerals with a higher melting temperature and low sagging risk [44]. The hydrochar (250-30) showed low SI risk and medium FI risk compared to the raw material. The main reason for that was that during high-severity HTC, the K and Na-bonded minerals leached from the raw material, and as a result, their content was relatively reduced in the hydrochars [77,78]. Therefore, hydrochar produced at 250 °C for 30 min was observed to be better for fuel application in boilers than raw OP biomass.

### 3.4. Optimal HTC Process Conditions and Combustion Performance of Hydrochar

The experimental data from this study regarding hydrochar yield (HY), energy densification ratio (EDR), and energy yield (EY) were mathematically modeled using the Modde 6.0 software (Sartorius AG, Göttingen, Germany) and subjected to ANOVA analysis with a 95% confidence level. Table 5 shows the parameter values of the three mathematical models (Equation (1)), as well as the regression coefficients R^2^ and R^2^ adjustment. In all cases, the models were statistically significant (*p*-values < 0.0001), while the regression coefficient (R^2^), which represents the goodness-of-fit between experimental and predicted data, was higher than 0.965. Detailed ANOVA tables for all the responses are provided in Tables S12–S14 in Supplementary Information.

**Figure 5:**
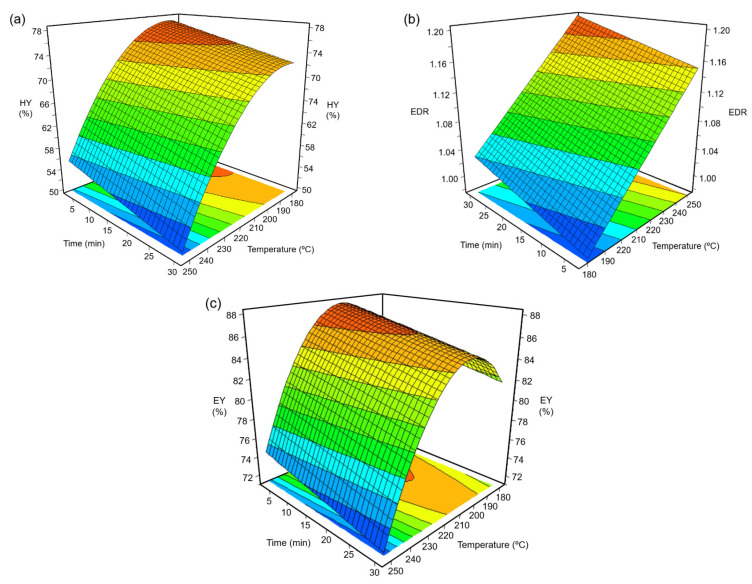
Response surface plots corresponding to (**a**) hydrochar yield, HY (%); (**b**) energy densification ratio, EDR; and (**c**) energy yield, EY (%).

The values of the parameters β_i_ from Table 5 reveal that both hydrochar yield and energy yield were negatively affected by two linear terms (*T* and *t*) and one quadratic term (*T* × *T*). On the other hand, the energy densification ratio was linearly and positively affected by temperature, reaction time, and the quadratic term (*T* × *T*) and negatively affected by the interaction term (*T* × *t*). In all cases, temperature had a much greater effect on the responses compared to reaction time. Figure 5 shows the 3D response surfaces and 2D contour plots generated by applying the three mathematical models for temperatures and reaction times in the ranges 180–250 °C and 2–30 min, respectively. The hydrochar yield (Figure 5a) ranged from a maximum value of 77.5% for treatment at 180 °C for 2 min to a minimum value of 49.9% for treatment at 250 °C for 30 min. The optimal conditions for achieving the highest HY value of 77.57% were 182.9 °C and 2.0 min. A HY of 53% was obtained in a previous study where the wet olive pomace underwent hydrothermal treatment at 260 °C for 60 min [22]. The decrease in HY with increasing temperature and reaction time could be explained by losses of aqueous extracts and holocellulose through hydrolysis and deoxygenation reactions. These reactions entail enrichment in the lignin of the pretreated solid, thereby increasing the energy densification ratio (from 1.07 to 1.40) in the hydrochars. The highest EDR value of 1.40 was obtained for the hydrochar produced at optimal conditions of 250.0 °C and 30 min.

In line with the present results, Missaoui et al. (2017) reported 250 °C for 30 min to be optimal HTC conditions that produced hydrochar with 1.20 EDR. In this study, the biomass–water ratio was varied by the use of distilled water and not inherent water. The 1/6 ratio was mentioned to be equivalent to 86% of the amount of moisture present [31]. Semaan et al. (2022) performed hydrothermal carbonization experiments with a biomass/water weight ratio of 1/3 at 180–250 °C for 0–60 min and reported 1.26 EDR for OP hydrochar [23]. The optimal HTC conditions for producing the hydrochar with the highest EY of 87.9% were 202.7 °C and 2.0 min. These optimal conditions were comparatively lower than the HTC conditions reported by earlier studies. Giminez et al. (2020) reported a maximum EY of 85% for hydrochar produced from two-phase olive pomace at optimal HTC conditions of 240 °C and 6 h [13]. Yay et al. (2021) found a maximum EY of 86% for three-phase olive pomace-derived hydrochar produced at 220 °C and 1 h [22]. 

### 3.5. TGA–DSC Analysis

**Figure 6:**
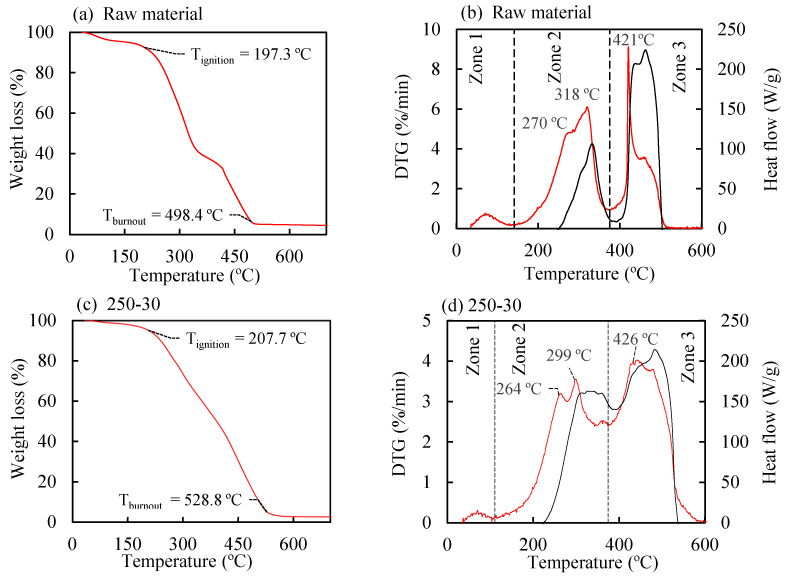
TG curves (**a**,**c**), DTG curves, and exothermic heat flow curves from DSC analyses (**b**,**d**) for the raw material and the hydrochar obtained at 250 °C for 30 min.

To compare the effect of hydrothermal treatment on the biomass combustion process, thermogravimetric analyses were conducted under an oxidizing atmosphere on the raw material and the hydrochar obtained at 250 °C for 30 min, which is the treated solid that exhibited the highest heating value. The weight loss and first derivative data (DTG) curves for the two analyzed biomasses under a heating rate of 10 °C·min^−1^ are shown in Figure 6. During the heating of the biomasses, various physical and chemical changes occurred in the materials; for example, at temperatures around 100 °C, the weight losses (Figure 6, left column) are attributed to the removal of water from the solids, while at the ignition temperatures (197.3 °C for the raw material and 207.7 °C for the hydrochar), the biomasses underwent significant weight reduction due to combustion. It is interesting to note that the higher T_i_ value for the hydrochar compared to the raw material (attributable to lower volatile matter content) could be beneficial during storage, implying a lower risk of self-combustion. The ignition temperatures of the olive pomace and the hydrochar were 498.4 °C and 528.8 °C, respectively, and above these temperatures, the solid weight remained practically constant. The increase in T_b_ (burnout temperature) in hydrothermally treated biomasses has been informed in previous studies, where it has been associated with the increase in fixed carbon in the materials [79]. Residence times for combustion could be determined from the difference between T_b_ and T_i_: 30.1 min for the raw material and 32.1 min for the hydrochar 250-30.

On the other hand, through the DTG curves (Figure 6, right column–red curves), three temperature regions were established where biomass underwent changes: Zone 1, or drying zone; Zone 2, where primarily the volatilization of organic compounds and their combustion in a homogeneous phase occurred; and Zone 3, where the solid materials (chars) generated at lower temperatures burned. For some biomasses, the thermal decomposition of cellulose occurs at temperatures slightly higher than that of hemicellulose [80,81]. For this reason, signals related to the loss of hemicellulose and cellulose could be distinguished in Zone 2 for both the raw material (shoulder at 270 °C and peak at 318 °C, respectively, Figure 6b) and the hydrochar (peaks at 264 °C and 299 °C, respectively, Figure 6d). It is interesting to note that hydrothermal treatment globally reduces the DTG signals in Zone 2, which could be explained by the decrease in holocellulose content in the treated biomass. This same reason could explain, in Zone 3, the significant reduction suffered by the DTG peak for the raw material at 421 °C (8.79%/min) when compared to that of the hydrochar at 426 °C (3.89%/min). In this regard, it is worth noting that these signals could be related to the combustion of char fractions derived from holocellulose. Based on the previous information, it can be established that hydrothermal treatment transforms the original biomass into a material with a more uniform combustion rate, reducing the presence of pronounced DTG peaks as well as the signal drop that occurs between combustion Zones 1 and 2 for the raw material.

Regarding the energy flows obtained from the DSC analyses (Figure 6, right column–black curves), the data reveal how hydrothermal treatment causes a significant shift of signals from Zone 3 to Zone 2, thus resulting in a more homogeneous energy material capable of generating similar amounts of thermal energy in both combustion regions.

## 4. Conclusions

The present work demonstrated that the hydrothermal carbonization of industrial two-phase olive pomace slurry produced hydrochar with promising fuel characteristics. The optimal conditions for producing the hydrochar with the highest energy yield (87.9%) were 202.7 °C and 2.0 min. The molecular structure of hydrochar was found to be mainly composed of heteroaromatic moieties associated with acid-insoluble lignin fractions and a limited amount of crystalline cellulose and other aliphatic fragments. Acid-insoluble lignin and fixed carbon content enrichment were identified as decisive factors in producing hydrochar with a high heating value, energy density, and better combustibility performance. Hydrochars also exhibited lower slagging–fouling risks than raw olive pomace biomass. These findings accentuated the idea that a lower HTC temperature (around 200 °C) could be used for the production of hydrochar in a more energy-efficient and cost-effective manner for its wider application as a biofuel. 

## Figures and Tables

**Table 1:** Methods for the calculation of slagging and fouling indices.

Indicator	Equations	Slagging and Fouling Risks		
		Low	Medium	High
Alkali index (AI)	$AI=\frac{{Na}_{2}O+K_{2}O}{HHV}$	<0.17	0.17–0.34	>0.34
Base-to-acid ratio (B/A)	$B/A=\frac{{Fe}_{2}O_{3}+{CaO+MgO+{Na}_{2}O+K}_{2}O+P_{2}O_{5}}{SiO_{2}+{Al}_{2}O_{3}+TiO_{2}}$	-	-	-
Slagging index (SI)	SI=B/A×S	<0.6	0.6–2.0	2.0–2.6
Fouling index (FI)	$FI=B/A\times ({Na}_{2}O+K_{2}O)$	0.2	0.2–0.5	0.51.0
Slag viscosity index (SVI)	$SVI=SiO_{2}\times 100$	>72	65–72	<65
Bed agglomeration index (BAI)	$BAI=\frac{{Fe}_{2}O_{3}}{{Na}_{2}O+K_{2}O}$	-	-	-

Note: Units used for the calculation of AI (Kg·GJ^−1^) were HHV in GJ·Kg^−1^ and Na_2_O and K_2_O in Kg. Mineral content in % was used for the calculation of B/A, SI, FI, SVI, and BAI.

**Table 2:** Yield, acid-insoluble lignin, extractives, and proximate characteristics of hydrochars on an oven-dried basis.

Samples	HY (%)	AIL (%)	E (%)	Ash (%)	VM (%)	FC (%)	Fuel Ratio	FCI
OP	-	36.82 ± 2.58	11.03 ± 0.86	4.25 ± 0.14	80.32 ± 1.41	15.43 ± 1.54	0.19	0.00
180-2	77.34	58.54 ± 0.07	18.04 ± 0.07	3.92 ± 0.04	77.60 ± 0.06	18.48 ± 0.10	0.24	0.11
180-16	75.31	58.22 ± 0.01	16.00 ± 0.15	3.86 ± 0.08	77.91 ± 0.19	18.23 ± 0.26	0.23	0.10
180-30	72.22	61.39 ± 0.05	18.19 ± 0.15	3.88 ± 0.15	78.20 ± 1.36	17.92 ± 1.51	0.23	0.09
215-2	74.01	63.73 ± 0.08	20.32 ± 0.01	3.83 ± 0.13	76.16 ± 1.19	20.01 ± 1.32	0.27	0.18
215-16	69.34	64.43 ± 0.29	19.41 ± 0.07	3.74 ± 0.04	74.94 ± 0.84	21.32 ± 0.80	0.28	0.21
215-16	66.33	65.10 ± 0.05	21.18 ± 0.67	3.86 ± 0.02	74.20 ± 1.13	21.94 ± 1.15	0.30	0.23
215-16	71.65	64.64 ± 0.12	19.17 ± 0.30	3.70 ± 0.12	73.31 ± 0.66	23.07 ± 0.66	0.31	0.27
215-30	67.99	66.49 ± 0.28	20.41 ± 0.23	3.78 ± 0.01	72.98 ± 0.13	23.24 ± 0.14	0.32	0.28
250-2	54.47	72.53 ± 0.34	25.84 ± 0.04	3.52 ± 0.27	70.65 ± 0.37	25.83 ± 0.64	0.37	0.37
250-16	52.66	73.96 ± 0.16	23.14 ± 0.31	3.40 ± 0.02	69.55 ± 0.33	27.05 ± 0.31	0.39	0.42
250-30	50.19	76.01 ± 0.35	24.67 ± 0.07	2.78 ± 0.05	69.35 ± 0.88	27.87 ± 0.83	0.40	0.45

Note: Values are presented based on the duplicate analysis.

**Table 3:** Ultimate and energetic characteristics of hydrochars on an oven-dried basis.

Samples	C (%)	H (%)	N (%)	O (%)	CDF	HHV (MJ·Kg^−1^)	EDR	EY (%)
OP	57.44 ± 0.98	7.39 ± 0.14	1.64 ± 0.35	29.32 ± 1.30	1.00	22.92 ± 0.26	1.00	100.00
180-2	59.80 ± 0.70	7.55 ± 0.08	1.47 ± 0.05	27.26 ± 0.82	1.04	24.62 ± 0.01	1.07	83.07
180-16	60.47 ± 0.14	7.61 ± 0.04	1.64 ± 0.07	26.42 ± 0.25	1.05	25.57 ± 0.00	1.12	84.01
180-30	61.43 ± 0.75	7.67 ± 0.04	1.66 ± 0.08	25.36 ± 0.88	1.07	26.29 ± 0.01	1.15	82.82
215-2	65.42 ± 0.10	7.92 ± 0.07	1.79 ± 0.08	21.03 ± 0.08	1.14	27.34 ± 0.02	1.19	88.26
215-16	64.95 ± 1.32	7.70 ± 0.02	1.72 ± 0.13	22.01 ± 1.38	1.13	27.87 ± 0.00	1.22	84.30
215-16	65.78 ± 0.04	7.76 ± 0.05	1.71 ± 0.02	21.06 ± 0.01	1.15	27.92 ± 0.00	1.22	84.63
215-16	64.00 ± 1.37	7.71 ± 0.18	1.63 ± 0.10	22.79 ± 1.65	1.11	27.84 ± 0.00	1.21	87.00
215-30	65.61 ± 1.87	7.79 ± 0.15	1.74 ± 0.15	21.07 ± 2.17	1.14	28.18 ± 0.04	1.23	83.58
250-2	68.50 ± 0.40	7.89 ± 0.14	1.79 ± 0.08	18.42 ± 0.34	1.19	31.34 ± 0.01	1.37	74.47
250-16	71.26 ± 0.03	8.16 ± 0.13	1.80 ± 0.04	15.26 ± 0.12	1.24	31.89 ± 0.01	1.39	73.25
250-30	71.83 ± 0.73	7.98 ± 0.12	1.90 ± 0.01	15.51 ± 0.87	1.25	32.20 ± 0.01	1.40	70.49

Note: Values are presented based on the duplicate analysis.

**Table 4:** Mineral composition (average values in percentage (%)), slagging and fouling indices of raw material, and hydrochars on an oven-dried basis.

Samples	Na_2_O	MgO	Al_2_O_3_	SiO_2_	P_2_O_5_	K_2_O	CaO	Fe_2_O_3_	AI	B/A	SI	FI	SVI	BAI
OP	0.28	0.15	0.06	0.12	0.18	2.41	0.64	0.11	1.17	20.59	0.21	55.43	12.07	0.04
180-2	0.33	0.07	0.03	0.02	0.68	1.40	0.68	0.03	0.70	60.10	0.60	103.71	2.51	0.02
180-16	0.39	0.10	0.05	0.07	0.65	1.39	0.80	0.04	0.70	28.12	0.28	50.10	7.25	0.02
180-30	0.45	0.10	0.06	0.12	0.70	1.32	1.16	0.04	0.67	21.03	0.21	37.24	8.22	0.03
215-2	0.60	0.11	0.05	0.07	0.77	1.42	0.89	0.04	0.74	30.79	0.31	62.25	6.29	0.02
215-16	0.39	0.09	0.05	0.08	0.75	1.22	0.86	0.04	0.58	24.67	0.25	39.57	7.54	0.03
215-30	0.27	0.09	0.06	0.10	0.73	1.35	0.78	0.05	0.57	19.95	0.20	32.23	10.08	0.03
250-2	0.80	0.12	0.05	0.02	0.49	0.75	0.94	0.04	0.49	48.72	0.49	75.32	1.58	0.03
250-16	0.44	0.10	0.07	0.11	0.92	0.84	0.96	0.06	0.40	17.83	0.18	22.69	8.93	0.05
250-30	0.07	0.09	0.08	0.17	1.06	0.49	0.81	0.06	0.17	10.53	0.11	5.91	14.60	0.11

**Table 5:** Model parameters (β_i_), regression coefficients (R^2^ and R^2^ adjust), and F ratios for the models representing hydrochar yield (HY,%), energy densification ratio (EDR), and energy yield (EY) of the hydrochars.

Parameters	HY (%)	EDR	EY (%)
β_0_	69.9 ± 0.8	1.210 ± 0.000	85.50 ± 0.60
β_1_	−11.3 ± 0.7	0.107 ± 0.002	−5.28 ± 0.53
β_2_	−2.6 ± 0.7	0.019 ± 0.002	−1.49 ± 0.53
β_3_	−6.2 ± 1.0	0.022 ± 0.002	−7.50 ± 0.79
β_4_	NS	NS	NS
β_5_	NS	−0.005 ± 0.002	NS
R^2^	0.979	0.998	0.965
R^2^ adjust	0.970	0.997	0.951
F ratio	107.9	901.5	65.1

Note: Models were obtained from Equation (1). Data are shown with three significant figures. NS: no statistical significance.

## Data Availability

Extended and underlying dataset with title “Industrial Two-phase Olive Pomace Slurry-Derived Hydrochar Fuel for Energy Applications” for present publication are available on the Zenodo repository (https://doi.org/10.5281/zenodo.11353931) This dataset can be openly accessed under Creative Commons Attribution 4.0 International license, and includes following data files: (1) Dataset description.txt (provides abbreviations or codes of samples and their properties). (2) Extended data.xlsx (data files for the biochemical, proximate, ultimate, HHV, and mineral characterisitics of raw material (two-phase olive pomace slurry) and hydrochars. (3) 13C-NMR data.zip (raw data files for 13C-solid nuclear magnetic resonance analysis of raw material and hydrochars). (4) TGA-DTA data.zip (raw data for thermal gravimetric analysis of raw material and hydrochars). (5) FTIR data.zip (raw data for fourier transform infrared analysis of raw material and hydrochars).

## Supplementary Materials

The following supporting information can be downloaded at: https://www.mdpi.com/article/10.3390/polym16111529/s1, Table S1. Operational conditions assayed for hydrothermal carbonisation of olive pomace slurry. Table S2: NMR spectra peaks characteristics of raw olive pomace. Table S3: NMR spectra peaks characteristics of hydrochar produced at 180 °C-2 min. Table S4: NMR spectra peaks characteristics of hydrochar produced at 180 °C-16 min. Table S5: NMR spectra peaks characteristics of hydrochar produced at 180 °C-30 min. Table S6: NMR spectra peaks characteristics of hydrochar produced at 215 °C-2 min. Table S7: NMR spectra peaks characteristics of hydrochar produced at 215 °C-16 min. Table S8: NMR spectra peaks characteristics of hydrochar produced at 215 °C-30 min. Table S9: NMR spectra peaks characteristics of hydrochar produced at 250 °C-2 min. Table S10: NMR spectra peaks characteristics of hydrochar produced at 250 °C-16 min. Table S11: NMR spectra peaks characteristics of hydrochar produced at 250 °C-30 min. Table S12: ANOVA parameters calculated on basis of response surface experimental design for hydrochar yield. Table S13: ANOVA parameters calculated on basis of response surface experimental design for energy densification ratio (EDR). Table S14: ANOVA parameters calculated on basis of response surface experimental design for energy yield (EY).

## Nomenclature

OP = Olive pomace
HTC = Hydrothermal carbonisation
180-2 = Hydrochar produced at 180 °C with 2 min holding time
180-16 = Hydrochar produced at 180 °C with 16 min holding time
180-30 = Hydrochar produced at 180 °C with 30 min holding time
215-2 = Hydrochar produced at 215 °C with 2 min holding time
215-16 = Hydrochar produced at 215 °C with 16 min holding time
215-30 = Hydrochar produced at 215 °C with 30 min holding time
250-2 = Hydrochar produced at 250 °C with 2 min holding time
250-16 = Hydrochar produced at 250 °C with 16 min holding time
250-30 = Hydrochar produced at 250 °C with 30 min holding time
NMR = Nuclear magnetic resonance
FTIR = Fourier transform infrared spectroscopy
TGA = Thermogravimetric analysis
DSC = Differential scanning calorimetry
βi = Model parameters for response surface
Ti = Ignition temperature
Tb = Burnout temperature
E = Extractives obtained by hexane solvent extraction
AIL = Acid insoluble lignin
HY = Hydrochar yield
VM = Volatile matter
FC = Fixed carbon
FCI = Fixed carbon index
CDF = Carbon densification factor
HHV = Higher heating value
EDR = Energy densification ratio
EY = Energy yield
AI = Alkali index
B/A = Base to acid ratio
SI = Slagging index
FI = Fouling index
SVI = Slag viscosity index
BAI = Bed agglomeration index
