# Supplementary material for: Industrial Two-Phase Olive Pomace Slurry-Derived Hydrochar Fuel for Energy Applications
Source: Polymers (Basel). 2024 May 29;16(11):1529. doi: 10.3390/polym16111529 (PMC11175087; doi:10.3390/polym16111529)
Supplement: Supplementary file 1 [file polymers-16-01529-s001.zip › polymers-3006370-supplementary.pdf]

Industrial Two-phase Olive Pomace Slurry-Derived Hydrochar Fuel for Energy Applications

Adnan Asad Karim [1,2,*], Mª Lourdes Martínez-Cartas [1,2,*] and Manuel Cuevas-Aranda [1,2]

1 Department of Chemical, Environmental and Materials Engineering, Science & Technology Campus (Linares), University of Jaén, Avda. de la Universidad s/n, 23700 Linares, Spain; mcuevas@ujaen.es
2 University Institute of Research on Olive and Olive Oils (INUO), University of Jaén, Campus de las Lagunillas s/n, 23071 Jaén, Spain
* Correspondence: aasad@ujaen.es (A.A.K.); lcartas@ujaen.es (M.L.M.C.)

Table of Contents

- Table S1. Operational conditions assayed for hydrothermal carbonisation of olive pomace slurry.
- Table S2: NMR spectra peaks characteristics of raw olive pomace.
- Table S3: NMR spectra peaks characteristics of hydrochar produced at 180 °C-2 min.
- Table S4: NMR spectra peaks characteristics of hydrochar produced at 180 °C-16 min.
- Table S5: NMR spectra peaks characteristics of hydrochar produced at 180 °C-30 min.
- Table S6: NMR spectra peaks characteristics of hydrochar produced at 215 °C-2 min.
- Table S7: NMR spectra peaks characteristics of hydrochar produced at 215 °C-16 min.
- Table S8: NMR spectra peaks characteristics of hydrochar produced at 215 °C-30 min.
- Table S9: NMR spectra peaks characteristics of hydrochar produced at 250 °C-2 min.
- Table S10: NMR spectra peaks characteristics of hydrochar produced at 250 °C-16 min.
- Table S11: NMR spectra peaks characteristics of hydrochar produced at 250 °C-30 min.
- Table S12: ANOVA parameters calculated on basis of response surface experimental design for hydrochar yield.
- Table S13: ANOVA parameters calculated on basis of response surface experimental design for energy densification ratio (EDR).
- Table S14: ANOVA parameters calculated on basis of response surface experimental design for energy yield (EY).

Table S1. Operational conditions assayed for hydrothermal carbonisation of olive pomace slurry.

**Samples (Temperature-time)**	T_R **(ºC)**		t_R **(min)**	
	Coded	Real	Coded	Real
**180-2**	– 1	180	– 1	2
**180-16**	– 1	180	0	16
**180-30**	– 1	180	+ 1	30
**215-2**	0	215	– 1	2
**215-16**	0	215	0	16
**215-16**	0	215	0	16
**215-16**	0	215	0	16
**215-30**	0	215	+ 1	30
**250-2**	+ 1	250	– 1	2
**250-16**	+ 1	250	0	16
**250-30**	+ 1	250	+ 1	30

Note: T_R = reaction temperature; t_R = reaction time (holding time at T_R).

Table S2: NMR spectra peaks characteristics of raw olive pomace.

Peak Fit	Frequency		Width		Intensity	Area
	ppm	Hz	ppm	Hz		
1	171.732	21596.60	4.05657	510.145	1.815	117.972
STD	0.108	13.56	0.34704	43.643	0.090	
2	152.207	19141.18	4.52306	568.810	1.380	100.023
STD	0.190	23.92	0.55341	69.595	0.117	
3	135.027	16980.65	16.01611	2014.151	0.758	194.526
STD	0.361	45.44	1.30294	163.855	0.034	
4	104.220	13106.51	3.62799	456.248	6.792	394.784
STD	0.014	1.77	0.04037	5.076	0.053	
5	87.565	11012.01	3.29508	414.382	2.257	119.142
STD	0.073	9.21	0.23233	29.218	0.099	
6	82.076	10321.68	6.86324	863.105	3.478	382.418
STD	0.219	27.49	0.86993	109.400	0.212	
7	72.875	9164.60	6.07293	763.718	14.976	1457.012
STD	0.051	6.36	0.18180	22.863	0.252	
8	63.609	7999.36	6.00160	754.748	6.147	590.989
STD	0.045	5.68	0.17116	21.525	0.093	
9	55.895	7029.17	6.01917	756.958	3.887	374.840
STD	0.069	8.67	0.22755	28.616	0.088	
10	38.843	4884.81	12.74593	1602.900	0.798	163.045
STD	0.472	59.41	1.55251	195.240	0.030	
11	33.836	4255.20	5.53027	695.475	1.086	96.209
STD	0.098	12.28	0.48740	61.295	0.036	
12	29.164	3667.57	3.71040	466.612	1.689	100.426
STD	0.086	10.80	0.29330	36.884	0.078	
13	20.643	2595.96	4.77532	600.534	2.558	195.725
STD	0.059	7.43	0.18748	23.577	0.064	

Table S3: NMR spectra peaks characteristics of hydrochar produced at 180 °C-2 min.

Peak Fit	Frequency		Width		Intensity	Area
	ppm	Hz	ppm	Hz		
1	172.160	21650.52	3.73845	470.140	1.267	75.894
STD	0.205	25.79	0.65463	82.325	0.130	
2	144.906	18223.03	8.43497	1060.763	1.898	256.465
STD	0.149	18.75	0.46415	58.371	0.068	
3	130.530	16415.14	13.79675	1735.049	1.495	330.548
STD	0.129	16.22	0.50521	63.534	0.026	
4	109.728	13799.16	13.72632	1726.191	1.049	230.772
STD	2.138	268.91	7.72587	971.588	0.331	
5	88.068	11075.23	2.32591	292.501	3.903	145.452
STD	0.037	4.63	0.11035	13.877	0.124	
6	82.361	10357.48	6.86983	863.934	3.195	351.690
STD	0.040	5.02	0.17946	22.569	0.036	
7	74.043	9311.48	3.95496	497.367	12.484	791.030
STD	0.024	3.04	0.07146	8.987	0.098	
8	71.750	9023.13	3.10175	390.069	15.458	768.126
STD	0.026	3.24	0.08769	11.027	0.224	
9	64.024	8051.56	5.49034	690.452	5.924	521.093
STD	0.061	7.72	0.22289	28.030	0.134	
10	55.577	6989.18	6.21325	781.364	4.664	464.230
STD	0.062	7.76	0.22732	28.587	0.094	
11	46.804	5885.94	14.70763	1849.600	1.043	245.873
STD	0.387	48.70	2.77010	348.361	0.042	
12	38.767	4875.21	8.74197	1099.371	1.527	213.819
STD	0.133	16.66	0.68547	86.203	0.038	
13	32.562	4094.96	11.76547	1479.600	1.877	353.874
STD	0.677	85.11	4.58518	576.622	0.171	
14	25.540	3211.91	18.62945	2342.798	1.892	564.615

STD	0.387	48.72	2.19751	276.355	0.040	
15	13.984	1758.60	7.84364	986.399	0.230	28.944
STD	4.002	503.23	14.82455	1864.303	0.148	

Table S4: NMR spectra peaks characteristics of hydrochar produced at 180 °C-16 min.

Peak Fit	Frequency		Width		Intensity	Area
	ppm	Hz	ppm	Hz		
1	172.570	21702.07	2.58352	324.897	1.007	41.659
STD	0.193	24.25	0.60962	76.664	0.147	
2	146.255	18392.69	6.35309	798.950	1.892	192.577
STD	0.134	16.88	0.43125	54.233	0.078	
3	137.945	17347.69	7.84370	986.406	0.490	61.553
STD	0.786	98.85	3.36348	422.983	0.067	
4	134.109	16865.21	4.25550	535.162	1.213	82.715
STD	0.063	7.95	0.31243	39.291	0.034	
5	130.403	16399.14	10.78501	1356.299	1.160	200.443
STD	0.304	38.22	2.14580	269.851	0.032	
6	125.193	15744.02	12.74586	1602.891	0.632	128.982
STD	0.999	125.59	5.45503	686.012	0.049	
7	116.378	14635.49	14.05986	1768.137	0.733	165.187
STD	0.265	33.28	1.48910	187.266	0.022	
8	104.447	13134.97	2.53204	318.423	8.354	338.894
STD	0.015	1.91	0.04347	5.466	0.101	
9	88.124	11082.29	2.32859	292.839	4.083	152.311
STD	0.038	4.72	0.11082	13.936	0.132	
10	82.487	10373.41	7.98927	1004.713	2.989	382.521
STD	0.079	9.93	0.37324	46.938	0.051	
11	74.105	9319.33	3.99962	502.983	12.529	802.839
STD	0.023	2.84	0.06741	8.477	0.087	
12	71.775	9026.31	3.17550	399.344	15.457	786.376
STD	0.027	3.37	0.09203	11.573	0.231	
13	63.956	8042.97	5.31786	668.763	6.226	530.410
STD	0.064	8.00	0.22868	28.758	0.151	
14	55.697	7004.38	5.50431	692.210	4.733	417.324

STD	0.051	6.43	0.16303	20.503	0.088	
15	38.159	4798.85	10.78501	1356.299	1.193	206.148
STD	0.427	53.72	1.21252	152.484	0.046	
16	33.227	4178.51	7.97842	1003.349	1.617	206.660
STD	0.187	23.51	1.05228	132.333	0.045	
17	29.585	3720.60	4.80652	604.458	2.627	202.307
STD	0.108	13.56	0.39411	49.563	0.104	
18	21.652	2722.91	9.87880	1242.336	1.677	265.338
STD	0.179	22.48	1.07081	134.662	0.051	

Table S5: NMR spectra peaks characteristics of hydrochar produced at 180 °C-30 min.

Peak Fit	Frequency		Width		Intensity	Area
	ppm	Hz	ppm	Hz		
1	172.024	21633.32	2.59795	326.713	1.114	46.349
STD	0.242	30.45	0.70863	89.115	0.154	
2	146.334	18402.63	4.90216	616.484	2.398	188.349
STD	0.118	14.82	0.34104	42.888	0.116	
3	129.471	16282.04	16.66848	2096.191	1.270	339.144
STD	0.332	41.73	1.59644	20.765	0.047	
4	116.405	14638.87	11.76555	1479.609	0.959	180.848
STD	0.202	25.37	0.90527	113.844	0.032	
5	104.397	13128.74	2.55541	321.363	8.298	339.731
STD	0.018	2.20	0.5017	6.309	0.114	
6	88.033	11070.90	2.45958	309.311	4.192	165.181
STD	0.044	5.48	0.12971	16.313	0.149	
7	82.315	10351.77	6.23205	783.729	2.911	290.619
STD	0.047	5.94	0.19905	25.033	0.042	
8	74.025	9309.20	3.76077	472.946	12.038	725.276
STD	0.032	4.00	0.09563	12.026	0.134	
9	71.686	9015.13	2.98170	374.971	15.419	736.569
STD	0.026	3.25	0.08793	11.058	0.242	
10	63.985	8046.61	5.59257	703.310	5.911	529.576
STD	0.063	7.97	0.23073	29.016	0.135	
11	55.531	6983.42	5.27044	662.799	5.051	426.485
STD	0.058	7.26	0.17941	22.562	0.111	
12	28.348	3564.99	22.55129	2836.001	2.507	905.656
STD	1.073	134.89	2.44836	307.901	0.080	
13	24.675	3103.07	10.05490	1264.482	1.983	319.482
STD	0.142	17.90	1.09736	138.002	0.040	

Table S6: NMR spectra peaks characteristics of hydrochar produced at 215 °C-2 min.

Peak Fit	Frequency		Width		Intensity	Area
	ppm	Hz	ppm	Hz		
1	174.204	21907.46	7.56784	951.715	1.026	124.450
STD	0.341	42.83	1.09096	137.197	0.074	
2	151.569	19061.03	2.70974	340.771	1.285	55.783
STD	0.229	28.78	0.70320	88.433	0.217	
3	145.956	18355.05	4.90216	616.484	3.751	294.60
STD	0.167	21.06	0.60408	75.967	0.259	
4	137.165	17249.58	10.78493	1356.289	1.941	335.287
STD	0.491	61.75	2.58355	324.902	0.149	
5	129.919	16338.33	10.78509	1356.309	2.998	517.980
STD	0.277	34.81	1.07064	134.641	0.123	
6	115.156	14481.71	8.82416	1109.707	1.926	272.307
STD	0.269	33.81	1.00328	126.170	0.117	
7	104.501	13141.80	2.69692	339.159	8.564	370.007
STD	0.026	3.30	0.07551	9.496	0.167	
8	87.942	11059.33	1.84519	232.047	5.589	165.205
STD	0.040	4.98	0.11614	14.605	0.241	
9	83.358	10482.92	5.01954	631.247	2.793	224.604
STD	0.046	5.79	0.16987	21.362	0.047	
10	73.964	9301.59	3.43341	431.778	12.191	670.554
STD	0.029	3.70	0.09413	11.837	0.167	
11	71.483	8989.58	2.70290	339.910	15.770	682.886
STD	0.026	3.22	0.08575	10.783	0.273	
12	64.044	8054.05	5.60969	705.462	6.197	556.919
STD	0.079	9.87	0.29474	37.066	0.175	
13	55.399	6966.86	6.11023	768.408	6.357	622.282
STD	0.054	6.83	0.19856	24.971	0.114	
14	46.956	5905.14	8.82408	1109.697	2.123	300.140

STD	0.472	59.32	2.07351	260.760	0.123	
15	42.839	5387.36	19.60995	2466.104	2.295	721.100
STD	1.629	204.89	26.53945	3337.543	0.150	
16	38.119	4793.77	7.95891	1000.895	3.894	496.553
STD	0.084	10.56	0.53525	67.312	0.068	
17	33.460	4207.86	6.79388	854.383	4.695	511.036
STD	0.056	7.01	0.39341	49.475	0.059	
18	29.455	3704.14	3.47283	436.735	8.124	451.992
STD	0.041	5.11	0.15861	19.947	0.190	
19	25.152	3163.05	7.73802	973.116	4.638	574.955
STD	0.122	15.33	0.52233	65.687	0.102	

Table S7: NMR spectra peaks characteristics of hydrochar produced at 215 °C-16 min.

Peak Fit	Frequency		Width		Intensity	Area
	ppm	Hz	ppm	Hz		
1	195.500	24585.69	13.72647	1726.211	1.094	240.495
STD	0.855	107.47	2.39267	300.897	0.059	
2	176.123	22148.81	9.80462	1233.008	2.077	326.287
STD	0.156	19.64	0.48895	61.489	0.067	
3	146.709	18449.75	10.73402	1349.887	4.571	786.005
STD	0.071	8.93	0.23031	28.963	0.061	
4	130.030	16352.31	9.80462	1233.008	2.901	455.720
STD	0.340	42.78	1.09072	137.166	0.203	
5	104.960	13199.56	2.76214	347.361	9.125	403.778
STD	0.059	7.37	0.16661	20.953	0.388	
6	88.237	11096.53	2.22926	280.346	5.976	213.419
STD	0.027	3.38	0.08259	10.387	0.144	
7	83.790	10537.18	7.55189	949.709	2.943	356.049
STD	0.140	17.64	0.60793	76.452	0.090	
8	74.324	9346.77	3.39895	427.445	13.458	732.807
STD	0.023	2.86	0.07360	9.255	0.149	
9	71.681	9014.42	2.46845	310.427	15.979	631.910
STD	0.034	4.26	0.11247	14.144	0.420	
10	64.220	8076.17	5.63210	708.281	6.123	552.468
STD	0.089	11.16	0.33170	41.713	0.194	
11	55.569	6988.28	5.53332	695.859	6.537	579.455
STD	0.045	5.71	0.14435	18.153	0.108	
12	38.373	4825.71	11.76547	1479.600	2.813	530.200
STD	0.305	38.41	1.16421	146.409	0.121	
13	29.897	3759.75	8.52275	1071.802	5.591	763.360
STD	0.177	22.26	0.86520	108.805	0.195	
14	24.584	3091.68	7.38338	928.518	3.995	472.498
STD	0.092	11.59	0.53050	66.714	0.085	

Table S8: NMR spectra peaks characteristics of hydrochar produced at 215 °C-30 min.

Peak Fit	Frequency		Width		Intensity	Area
	ppm	Hz	ppm	Hz		
1	145.640	18315.39	9.88276	1242.833	3.339	528.706
STD	0.100	12.64	0.37219	46.805	0.055	
2	128.833	16201.80	12.74586	1602.891	2.733	558.071
STD	0.164	20.68	0.57824	72.718	0.071	
3	104.624	13157.33	2.81801	354.387	8.615	388.935
STD	0.045	5.63	0.12728	16.006	0.274	
4	88.069	11075.41	1.80291	226.730	5.165	149.175
STD	0.043	5.35	0.12651	15.909	0.245	
5	83.126	10453.79	4.40538	554.011	2.808	198.207
STD	0.036	4.56	0.12789	16.083	0.046	
6	74.121	9321.28	3.14475	395.477	12.461	627.776
STD	0.025	3.13	0.07977	10.031	0.163	
7	71.599	9004.19	2.65130	333.421	15.725	667.946
STD	0.024	2.97	0.07916	9.955	0.262	
8	64.370	8095.07	5.42335	682.028	5.924	514.712
STD	0.086	10.85	0.31481	39.590	0.190	
9	55.353	6961.08	6.38186	802.569	5.513	563.624
STD	0.090	11.31	0.28832	36.258	0.155	
10	32.813	4126.48	14.70763	1849.600	3.446	812.039
STD	0.637	80.06	1.36140	171.207	0.111	
11	29.289	3683.29	5.73517	721.242	5.760	529.201
STD	0.123	15.42	0.47988	60.349	0.220	

Table S9: NMR spectra peaks characteristics of hydrochar produced at 250 °C-2 min.

Peak Fit	Frequency		Width		Intensity	Area
	ppm	Hz	ppm	Hz		
1	146.188	18384.29	7.84370	986.406	5.593	702.807
STD	0.260	32.68	0.75752	95.264	0.219	
2	129.349	16266.66	14.70771	1849.609	4.796	1130.127
STD	0.241	30.33	0.89719	112.828	0.156	
3	104.813	13181.04	3.70437	465.854	6.888	408.786
STD	0.119	14.92	0.33973	42.724	0.442	
4	87.818	11043.80	1.65227	207.785	4.223	111.792
STD	0.054	6.84	0.15884	19.975	0.279	
5	82.985	10435.98	4.22231	530.989	2.291	154.996
STD	0.056	7.01	0.19392	24.387	0.060	
6	74.050	9312.42	3.02337	380.212	10.659	516.287
STD	0.021	2.70	0.07073	8.895	0.134	
7	71.411	8980.54	2.84085	357.259	13.446	611.944
STD	0.031	3.95	0.10576	13.300	0.272	
8	63.777	8020.48	7.44518	936.289	5.597	667.549
STD	0.086	10.82	0.37817	47.557	0.126	
9	55.019	6919.09	8.27031	1040.056	6.787	899.294
STD	0.131	16.43	0.43170	54.290	0.207	
10	29.314	3686.41	15.42534	1939.857	9.187	2270.393
STD	0.221	27.83	0.79056	99.419	0.200	

Table S10: NMR spectra peaks characteristics of hydrochar produced at 250 °C-16 min.

Peak Fit	Frequency		Width		Intensity	Area
	ppm	Hz	ppm	Hz		
1	145.697	18322.51	11.76555	1479.609	3.705	698.428
STD	0.266	33.50	0.84626	106.424	0.083	
2	128.904	16210.67	8.82416	1109.707	4.306	608.747
STD	0.656	82.49	1.14163	143.568	0.261	
3	127.220	15998.91	9.29778	1169.269	3.981	593.063
STD	0.210	26.37	2.93122	368.624	0.072	
4	125.243	15750.31	8.61715	1083.674	3.737	515.959
STD	0.368	46.25	4.12650	518.939	0.115	
5	122.798	15442.79	14.70771	1849.609	3.713	874.900
STD	0.528	66.41	2.58363	324.912	0.058	
6	114.765	14432.63	11.76555	1479.609	3.359	633.113
STD	0.143	18.03	0.73265	92.137	0.069	
7	104.404	13129.65	3.19556	401.866	7.402	378.930
STD	0.037	4.64	0.10752	13.521	0.172	
8	87.850	11047.77	1.33624	168.042	3.151	67.464
STD	0.060	7.58	0.17445	21.938	0.285	
9	83.318	10477.86	3.38384	425.545	1.196	64.833
STD	0.103	12.96	0.33491	42.117	0.073	
10	73.531	9247.12	3.00075	377.368	8.671	416.868
STD	0.074	9.28	0.23704	29.809	0.360	
11	71.317	8968.67	2.90218	364.972	11.821	549.619
STD	0.027	3.38	0.08805	11.073	0.175	
12	63.938	8040.66	6.16039	774.717	5.884	580.705
STD	0.077	9.72	0.29493	37.090	0.148	
13	55.066	6925.00	6.50826	818.464	7.370	768.442
STD	0.035	4.34	0.12612	15.860	0.079	
14	45.569	5730.71	15.86639	1995.322	2.873	730.199
STD	0.380	47.82	2.32145	291.941	0.048	
15	41.320	5196.28	11.76547	1479.600	3.476	655.197
STD	0.533	67.06	3.69276	464.393	0.185	
16	32.529	4090.84	11.04417	1388.890	6.871	1215.650
STD	0.207	25.97	0.63686	80.090	0.079	
17	29.336	3689.27	3.63607	457.264	12.838	747.856
STD	0.054	6.76	0.21541	27.090	0.379	
18	26.101	3282.39	15.03147	1890.324	6.944	1672.256
STD	0.393	49.40	1.20262	151.239	0.102	
19	14.373	1807.53	11.76547	1479.600	3.630	684.286
STD	0.351	44.17	1.81867	228.712	0.147	

Table S11: NMR spectra peaks characteristics of hydrochar produced at 250 °C-30 min.

Peak Fit	Frequency		Width		Intensity	Area
	ppm	Hz	ppm	Hz		
1	145.274	18269.33	8.23877	1036.089	4.614	608.955
STD	0.170	21.44	0.60631	76.249	0.191	
2	128.000	16096.99	20.34123	2558.068	4.697	1530.552
STD	0.119	14.99	0.53805	67.664	0.053	
3	105.859	13312.64	16.91811	2127.584	2.857	774.274
STD	0.495	62.28	1.99649	251.074	0.165	
4	87.847	11047.48	1.06445	133.863	1.853	31.594
STD	0.133	16.79	0.38379	48.265	0.466	
5	82.959	10432.78	3.65925	460.180	1.466	85.935
STD	0.112	14.04	0.36599	46.026	0.091	
6	73.844	9286.42	2.68077	337.127	6.042	259.510
STD	0.039	4.85	0.12566	15.803	0.160	
7	71.212	8955.52	3.29579	414.471	7.531	397.622
STD	0.049	6.15	0.16169	20.334	0.191	
8	62.718	7887.30	11.00073	1383.428	3.630	639.791
STD	0.170	21.33	0.98743	124.177	0.097	
9	54.728	6882.51	13.49971	1697.694	5.313	1149.118
STD	0.241	30.34	0.80704	101.491	0.135	
10	28.340	3564.01	15.02716	1889.783	9.503	2287.863
STD	0.153	19.18	0.55776	70.143	0.176	
11	13.122	1650.25	12.74593	1602.900	4.382	894.806
STD	1.210	152.11	3.52685	443.529	0.343	

Table S12: ANOVA parameters calculated on basis of response surface experimental design for hydrochar yield.

Energy yield	DF	SS	MS	F	P	SD
			(variance)			
Total	11	49569.4	4506.31			
Constant	1	48646.1	48646.1			
Total Corrected	10	923.348	92.3348			9.6091
Regression	3	903.796	301.265	107.861	0.000	17.357
Residual	7	19.5516	2.79308			1.67125
Lack of Fit	5	5.31871	1.06374	0.149477	0.961	1.03138
(Model Error)						
Pure Error	2	14.2329	7.11643			2.66766
(Replicate Error)						
N = 11	Q2 =	0.963	Cond. no. =	2.7386		
DF = 7	R2 =	0.979	Y-miss =	0		
	R2 Adj. =	0.970	RSD =	1.6713		

Table S13: ANOVA parameters calculated on basis of response surface experimental design for energy densification ratio (EDR).

Energy yield	DF	SS	MS	F	P	SD
			(variance)			
Total	11	16.8618	1.53289			
Constant	1	16.7404	16.7404			
Total Corrected	10	0.121355	0.0121355			0.110161
Regression	4	0.121153	0.0302884	901.509	0.000	0.174036
Residual	6	0.000201584	3.35974e-005			0.00579633
Lack of Fit	4	0.000193584	4.8396e-005	12.0986	0.078	0.00695673
(Model Error)						
Pure Error	2	8.00037e-006	4.00014e-006			0.00200003
(Replicate Error)						
N = 11	Q2 =	0.709	Cond. no. =	2.6539		
DF = 6	R2 =	0.998	Y-miss =	0		
Comp. = 1	R2 Adj. =	0.997	RSD =	0.0058		

Table S14: ANOVA parameters calculated on basis of response surface experimental design for energy yield (EY).

Energy yield	DF	SS	MS	F	P	SD
			(variance)			
Total	11	73279.4	6661.76			
Constant	1	73933.4	72933.4			
Total Corrected	10	345.938	34.5938			5.88165
Regression	3	333.958	111.319	65.0481	0.000	10.5508
Residual	7	11.9794	1.71134			1.30818
Lack of Fit	5	7.36461	1.47292	0.638351	0.704	1.21364
(Model Error)						
Pure Error	2	4.61477	2.30738			1.51901
(Replicate Error)						
N = 11	Q2 =	0.907	Cond. no. =	2.7386		
DF = 7	R2 =	0.965	Y-miss =	0		
	R2 Adj. =		RSD =	1.3082		
